# Regulación de los servicios de medicina nuclear: percepción de la problemática y desafíos para el manejo del cáncer en Colombia

**DOI:** 10.7705/biomedica.6123

**Published:** 2021-12-15

**Authors:** Eliana Marcela Murcia, Johana Andrea Lineros, Jairo Aguilera, Carlos Eduardo Granados, María Cristina Martínez, Nathaly Barbosa

**Affiliations:** 1 Grupo de Evaluación y Seguimiento de Servicios Oncológicos, Instituto Nacional de Cancerología, Bogotá, D.C., Colombia Grupo de Evaluación y Seguimiento de Servicios Oncológicos Instituto Nacional de Cancerología Bogotá D.C. Colombia; 2 Grupo de Medicina Nuclear, Instituto Nacional de Cancerología, Bogotá, D.C., Colombia Grupo de Medicina Nuclear Instituto Nacional de Cancerología Bogotá D.C. Colombia

**Keywords:** cáncer de tiroides, medicina nuclear, residuos radiactivos, radioisótopos, servicios de salud, Thyroid neoplasms, nuclear medicine, radioactive waste, radioisotopes, health services

## Abstract

**Introducción.:**

La modificación de las normas sobre medicina nuclear en Colombia ha afectado la administración de la terapia de yodo radioactivo en el tratamiento del cáncer de tiroides.

**Objetivos.:**

Determinar las áreas de acuerdo en torno al problema, los requisitos actuales y los nuevos exigidos en la normativa para el funcionamiento de los servicios de medicina nuclear.

**Materiales y métodos.:**

Se hizo un estudio Delphi de dos rondas con cada grupo de expertos, ‘clínicos’ y ‘de entidades reguladoras’. En la primera ronda se exploraron los puntos de vista sobre las implicaciones de la normativa en medicina nuclear y, en la segunda, se calificaron las declaraciones de la primera según su relevancia.

**Resultados.:**

La problemática de los servicios de medicina nuclear está relacionada con la claridad normativa, y la falta de sinergia y coherencia entre los organismos de inspección, vigilancia y control. Las exigencias del sistema de gestión de desechos requieren una alta inversión económica que puede influir en la oferta del servicio y repercutir en el control integral del cáncer de tiroides. Entre las necesidades presentes y futuras, se encuentran la unificación de criterios entre los auditores, la delimitación de funciones de los actuantes, la asistencia técnica para cumplir con la normativa, y la veeduría a los organismos de inspección, vigilancia y control por parte de los entes reguladores.

**Conclusión.:**

Los hallazgos del estudio sugieren que los servicios de medicina nuclear atraviesan un momento de múltiples desafíos institucionales, normativos y económicos, que ponen en riesgo el desarrollo y mantenimiento de la medicina nuclear en la atención oncológica.

## Abstract (en)

**Introduction:**

Colombia has modified the nuclear medicine norms that impact the administration of radioactive iodine therapy in the treatment of thyroid cancer.

**Objective:**

To identify the areas of agreement regarding the issue, as well as the current and emergent requirements associated with the normative for the operation of nuclear medicine services that have an impact on the care of patients with thyroid cancer in Colombia.

**Materials and methods:**

We conducted a two-round Delphi study for each expert, clinical, and regulatory group. The first round explored views on the implications of the regulations that apply to nuclear medicine. The second round rated the statements from the first round by their relevance.

**Results:**

The issues regarding nuclear medicine services were related to the normative clarity and the lack of synergy and coherence among inspection, surveillance, and control bodies. The demands on the waste management system require a high economic investment that can influence the service offer and have an impact on the integral control of thyroid cancer. Unification of the auditors’ criteria, delimitation of the acting agent functions, technical assistance to the services to comply with the normative, and the oversight of the inspection, surveillance, and control bodies by the regulatory entities are among the current and future needs.

**Conclusions:**

Our findings suggest that nuclear medicine services are going through a time of multiple institutional, regulatory, and economic challenges that put at risk the development and maintenance of nuclear medicine in cancer care.

La medicina nuclear utiliza radionúclidos con fines diagnósticos y terapéuticos para detectar y tratar tumores y enfermedades benignas. En el contexto de la oncología moderna, esta especialidad involucra el uso de elementos radiactivos para evaluar las funciones corporales, determinar el diagnóstico y el tratamiento, así como la conjunción de los dos (“teragnóstica”) ^1^. Hay una gran variedad de radiofármacos utilizados en la medicina nuclear, pero en la mayoría de los procedimientos se recurre al tecnecio 99m (Tc^99m^) y al yodo radioactivo (I^131^) ^2^.

El cáncer de tiroides es la neoplasia endocrina más común (1,0-1,5 %), y es la quinto más frecuentemente diagnosticada en mujeres, con un incremento continuo en su incidencia mundial en los últimos 30 años ^3^. En el cáncer diferenciado de tiroides, el tratamiento con I^131^ se administra con tres propósitos: como tratamiento ablativo para eliminar tejido tiroideo residual sano después de una tiroidectomía; como tratamiento adyuvante para tratar la enfermedad residual microscópica, y como dosis terapéutica para tratar la enfermedad macroscópica o metastásica ^4^. Dependiendo del tipo de tratamiento, los pacientes sometidos a terapia con I^131^ son hospitalizados en habitaciones especiales, con el fin de controlar la irradiación a otros pacientes, familiares y cuidadores ^5^.

Al igual que en muchos países, en Colombia las habitaciones utilizadas para la terapia con yodo radioactivo están ubicadas en instituciones especializadas de prestación de servicios de salud ^6^, las cuales están sujetas a inspecciones por parte de los organismos de inspección, vigilancia y control de la seguridad radiológica y nuclear. En el último quinquenio, las autoridades nacionales competentes en materia nuclear han modificado las normas que permiten prestar servicios de medicina nuclear debido a las exigencias en el otorgamiento de autorizaciones para el empleo de fuentes radiactivas y la gestión de los desechos radiactivos liberados en el suelo, los cuerpos de agua o los sistemas de recolección de aguas residuales por medio de la orina y las excretas del paciente o la descarga de desechos líquidos hospitalarios ^7^.

Dicha regulación está ajustada a las directrices del Organismo Internacional de Energía Atómica (OIEA), del cual Colombia es miembro. Para honrar los compromisos adquiridos con este organismo en torno a una seguridad nuclear que proteja la salud humana, el país estableció desde el 2002 un marco regulador fundamental para el uso seguro de material radiactivo ^8^. En el cuadro 1 se presentan las normas nacionales que rigen la protección y seguridad radiológicas, el sistema de categorización de las fuentes radiactivas, las autorizaciones e inspecciones para el empleo de fuentes radiactivas, las licencias de importación de material radiactivo, la gestión de desechos radiactivos, el reglamento para instalaciones nucleares y para el transporte seguro de material radiactivo, y la licencia para la prestación de servicios de dosimetría personal.

**Cuadro 1:** Normativa para los servicios de medicina nuclear

Año	Norma	Objetivo
2002	Resolución 181434	Reglamentó la protección y seguridad radiológica; constituye el marco regulador fundamental para el uso seguro de materiales radiactivos y nucleares.
2004	Resolución 181419	Establece los requisitos y el procedimiento para la expedición de la licencia de importación de todo tipo de material radiactivo.
2004	Resolución 181289	Establece los requisitos para la obtención de licencias para la prestación del servicio de dosimetría personal
2008	Resolución 180052	Adopta el sistema de categorización, cuyo fundamento descansa en el daño potencial que la radiación puede causar a la salud humana.
2010	Resolución 180005	Reglamento para la gestión de los desechos radiactivos en el territorio colombiano
2014	Resolución 90874	Establece los requisitos y condiciones mínimas que se deben cumplir para la obtención de los diferentes tipos de autorización, y se otorga al órgano regulador la potestad de vigilancia y control mediante inspecciones o auditorías reguladoras para verificar las condiciones de protección radiológica y seguridad física de las instalaciones.
2015	Resolución 4245	Establece los requisitos para obtener la certificación en buenas prácticas de elaboración de radiofármacos y se adopta el instrumento para su verificación.
2016	Resolución 41226	Establece los requisitos y procedimientos para la expedición de autorizaciones para el empleo de fuentes radiactivas y de inspección de las instalaciones radiactivas.
2016	Resolución 41178	Por la cual se modifica la Resolución 180005 de 2010, la cual reglamenta la gestión de los desechos radiactivos en el territorio colombiano.
2018	Resolución 482	Reglamenta el uso de equipos generadores de radiación ionizante, su control de calidad y la prestación de servicios de protección radiológica.
2019	Resolución 3100	Define los procedimientos y condiciones de inscripción de los prestadores de servicios de salud y de habilitación de los servicios de salud.

Las modificaciones más importantes de la norma, que han generado reacciones de parte de los involucrados en la práctica de la medicina nuclear, están en la Resolución 180005, además de la contenida en la Resolución 41178, ambas emitidas por el Ministerio de Minas y Energía. En ellas, se establecen los niveles permitidos para vertidos líquidos a alcantarillas, ríos y otras grandes masas de agua ^9^^-^^10^. Estos estándares se armonizaron con respecto a las tasas de vertidos establecidas en otros países con actividades nucleares y radiactivas equiparables a las de Colombia. Entre los radionúclidos regulados, está el I^131^, cuya tasa de emisión mensual se fijó en una concentración de actividad de 1,90 bq/L, con un límite de emisión anual de 1,00E+07 bq/año ^10^. Estos límites incluyen la descarga de la actividad radiactiva derivada de la atención de los pacientes hospitalizados en las instituciones y de quienes recibieron el tratamiento de cáncer de tiroides con I^131^.

En este contexto, el objetivo del estudio fue establecer las áreas de acuerdo e incertidumbre frente a la problemática, los requisitos actuales y los que surgirán con las normas de funcionamiento de los servicios de medicina nuclear que repercuten en la atención del cáncer de tiroides en Colombia.

## Materiales y métodos

Se empleó el método Delphi para obtener la opinión de los expertos mediante cuestionarios virtuales con garantía de anonimato. Se construyó un marco de muestreo a partir de los servicios habilitados y se invitó a participar a 50 profesionales de la salud y a 24 funcionarios de entes reguladores provenientes de los 22 departamentos que contaban con servicios de medicina nuclear en ese momento. Cada uno de los participantes recibió un enlace a encuestas individualizadas realizadas en el cuarto trimestre del 2019 y el primer trimestre del 2020. El estudio constó de dos fases: preparatoria y de consulta.

### Fase preparatoria: selección de expertos

Se determinaron dos grupos de expertos: los ‘clínicos’ y los ‘reguladores’. Se determinaron los perfiles de los potenciales integrantes, quienes fueron contactados por correo electrónico para confirmar su colaboración. El grupo de expertos clínicos incluyó a profesionales con labores asistenciales relacionadas con la medicina nuclear en clínicas u hospitales públicos y privados del país. En el grupo de expertos reguladores participaron miembros de organizaciones involucradas o partes interesadas en la dinámica de funcionamiento, regulación y normativa de la medicina nuclear en Colombia. La decisión de participar fue voluntaria.

### Fase de consulta: rondas 1 y 2

En la ronda 1 se utilizó un cuestionario con enunciados y preguntas abiertas y semicerradas para que los participantes tuvieran libertad de plasmar sus puntos de vista en torno a la problemática de los servicios de medicina nuclear con repercusiones en la atención del cáncer de tiroides en el país. A los expertos se les interrogó sobre la normativa y la coherencia entre el sistema de salud, la gestión de desechos radiactivos líquidos de la terapia con yodo radioactivo, y el impacto en la atención de los pacientes con cáncer de tiroides. El período de respuesta de la encuesta fue de seis semanas y se envió un recordatorio a los invitados en la última semana. Se abarcaron temas relacionados con el diagnóstico y el tratamiento.

En la ronda 2 se tabularon y se categorizaron por temas las respuestas de la primera ronda para luego ser analizadas por los autores. Cada experto recibió los resultados del análisis estadístico de las respuestas grupales, con las declaraciones y consensos de la primera ronda. A partir de allí, se solicitó la confirmación de las respuestas grupales y la ratificación de algunas opiniones. Además de las declaraciones de consenso, se incluyeron dos preguntas abiertas, y se empleó una escala Likert para evaluar la opinión de los expertos, con el fin de capturar información adicional y detectar los elementos relacionados con el grado de desacuerdo o acuerdo previamente manifestado.

Para el análisis cualitativo, se empleó el programa Nvivo, versión 10, con codificación abierta según las categorías planteadas por el grupo investigador, con lo que se generaron nodos para el análisis contextual de los diferentes tipos de retos y de las referencias más frecuentes en el discurso de los expertos, y, por último, se consolidó, unificó y tabuló la información más relevante. Los datos cuantitativos se evaluaron en Excel.

## Resultados

### Expertos clínicos

**Figura 1:**
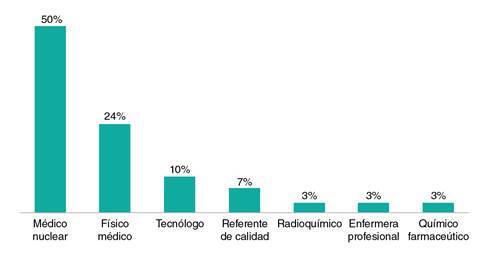
Profesiones de los expertos clínicos participantes

Participaron 30 profesionales, mínimo uno por departamento, que laboraban en servicios de medicina nuclear de consulta externa especializada o de apoyo diagnóstico y complementación terapéutica (figura 1).

En el cuadro 2 se presentan las respuestas obtenidas en la primera y la segunda ronda, relacionadas con el grado de acuerdo entre los expertos clínicos frente a los diez enunciados planteados por el equipo investigador.

**Cuadro 2:** Resultados de los expertos clínicos en la primera y segunda rondas del método Delphi

Enunciado	Ronda 1			Ronda 2	
	Desacuerdo %	Ni acuerdo ni desacuerdo %	Acuerdo %	Desacuerdo %	Acuerdo %
1. La normativa exigida para los servicios de medicina nuclear emitida por el Ministerio de Minas y Energía es clara y no permite interpretaciones.	40,0	23,3	36,7	76,7	23,3
2. Existe coherencia entre los parámetros técnicos regulados por la normativa y los requisitos internos para el funcionamiento de su servicio de medicina nuclear.	23,3	36,7	40,0	20,0	80,0
3. Los sistemas de gestión de desechos radiactivos líquidos exigidos en la Resolución 180005 de 2010 y su modificación 41178 de 2016, contribuyen a mejorar la calidad del agua de los ríos y grandes masas de agua.	26,7	23,3	50,0	16,7	83,3
4. La implementación de un sistema de gestión de desechos radiactivos líquidos implica una inversión monetaria adicional y disponibilidad de infraestructura que genera la disminución en la oferta de servicios que administran yodoterapias.	6,7	16,7	76,6	3,3	96,7
5. La normativa exigida para los servicios de medicina nuclear dictados por el Ministerio de Minas y Energía en cuanto al sistema de gestión de desechos radiactivos líquidos, es consecuente con la realidad del Sistema General de Seguridad Social en Salud.	53,3	33,4	13,3	90,0	10,0
6. Existe sinergia y coherencia entre los diferentes entes que realizan inspección, vigilancia y control de los servicios de medicina nuclear.	63,3	26,7	10,0	90,0	10,0
7. El estado debería asesorar y acompañar a los servicios de medicina nuclear para lograr el cumplimiento de la normativa exigida por el Ministerio de Minas y Energía, y por el Ministerio de Salud y Protección Social.	10,0	13,3	76,7	^_^	100
8. La oportunidad de la atención de pacientes con cáncer de tiroides que requieren terapias con I131 se ha visto afectada como consecuencia de la normativa vigente.	16,7	3,3	80,0	^_^	100
9. Las exigencias de los entes reguladores impactan en la prestación de los servicios de medicina nuclear de cara al control integral del cáncer de tiroides.	13,3	10,0	76,7	^_^	100
10. Existen dificultades para la aprobación de tirotropina alfa en pacientes con cáncer de tiroides que lo requieren.	33,3	16,7	50,0	16,7	83,3

Escala de Likert: Acuerdo: totalmente de acuerdo (5) y de acuerdo (4); ni acuerdo ni descuerdo (3); desacuerdo: desacuerdo (2) y totalmente en desacuerdo (1)

En cuanto a la normativa y el funcionamiento de los servicios de medicina nuclear, la mayoría del grupo de clínicos manifestó que había coherencia entre los parámetros técnicos regulados por la normativa y los requisitos internos de funcionamiento de los servicios de medicina nuclear, aunque resaltaron que en algunos aspectos eran excesivos. Sin embargo, los expertos concordaron en que la normativa exigida por el Ministerio de Minas y Energía a los servicios de medicina nuclear no es clara y permite múltiples interpretaciones, por lo cual concluyeron que no había sinergia ni coherencia entre los diferentes entes de inspección, vigilancia y control de estos servicios.

El panel estuvo de acuerdo en que las exigencias del Ministerio de Minas y Energía a los servicios de medicina nuclear en cuanto a la gestión de los desechos radiactivos líquidos no es consecuente con la realidad del sistema de salud colombiano, y que la implementación de un sistema de este tipo implica una alta inversión económica y una infraestructura sofisticada, lo cual podría afectar negativamente la oferta de los servicios que administran la terapia con yodo radioactivo. Sin embargo, estuvieron de acuerdo en que los requisitos de la Resolución 180005 del 2010 y su modificación en la Resolución 41178 del 2016 contribuyen a mejorar la calidad del agua de los ríos y las grandes masas de agua.

En este sentido, los miembros del panel acordaron unánimemente que el Estado debería asesorar y acompañar a los servicios de medicina nuclear para lograr el cumplimiento de la normativa exigida por los Ministerios de Minas y Energía y de Salud.

La opinión general frente a la atención de pacientes con cáncer que requieren la administración de terapias con I^131^, fue que la regulación ha afectado la oportunidad de las terapias y, por ende, el control integral del cáncer en el país.

Durante la primera ronda, se preguntó al grupo por los retos que implicaba el cumplimiento de la normativa y los requisitos vigentes de los organismos de inspección, vigilancia y control para los servicios de medicina nuclear del país. El panel de expertos emitió diversas opiniones. Se agruparon las declaraciones para generalizar situaciones que potencialmente podrían considerarse como desafíos, inquietudes y preocupaciones de los servicios de medicina nuclear en la atención del cáncer de tiroides. Esta información fue compartida en la segunda ronda y los participantes manifestaron estar de acuerdo con dichas afirmaciones (cuadro 3).

**Cuadro 3:** Declaraciones de los expertos sobre los desafíos en los servicios de medicina nuclear

Desafíos	Declaración de los expertos
Institucionales	a. Adecuar o remodelar los espacios donde funciona el servicio de medicina nuclear teniendo en cuenta la infraestructura disponible, el tipo de edificación y el costo elevado de las ampliaciones o remodelaciones, no va de la mano con las condiciones actuales de los servicios y del país. b. Implementar el sistema de vertidos para la gestión de desechos radiactivos líquidos como el I-131, es complejo y costoso, y los niveles de dispensa establecidos en la normativa son muy estrictos, de difícil cumplimiento y monitoreo. c. Disponibilidad de los tratamientos completos por parte de las EPS d. El acceso oportuno al material radiactivo se dificulta por falta de reactores nucleares en Colombia. e. Nuevo personal requerido para áreas específicas según la Resolución 3100 /2019 f. Formar y capacitar el personal de acuerdo con la realidad del país g. Baja demanda de tecnólogos en el área de medicina nuclear y capacitaciones a nivel nacional h. Altos estándares de exigencia a los servicios que solo brindan diagnóstico o terapias para hipotiroidismo
Normativos	a. Articular la normativa del Ministerio de Salud y Protección Social y el Ministerio de Minas y Energía, manteniendo la coherencia y evitando incongruencias y cambios frecuentes b. Unificar interpretaciones de la normativa por parte de los diferentes entes de control mediante el conocimiento de la norma, listas de chequeo apropiadas para los servicios y hojas de registro asertivas c. Implementación y cumplimiento de criterios normativos relacionados con el sistema de vertimiento y la radiofarmacia d. Mejorar la comunicación y prontitud en las respuestas frente a los requisitos exigidos a los prestadores de los servicios de medicina nuclear e. Realizar un acompañamiento por parte de los organismos del Estado a los servicios de medicina nuclear para facilitar el cumplimiento de los requisitos normativos
Económicos	a. Invertir en infraestructura, sistemas y equipos necesarios para el manejo de desechos líquidos representa un alto costo. b. La sostenibilidad financiera de los servicios de medicina nuclear se ve comprometida debido a que la inversión no está siendo recuperada mediante las tarifas cobradas a las EPS, ya que estas tarifas no han sido ajustadas por las EPS y los costos actuales no contemplan la maquila de los radiofármacos. c. Lograr que las EPS cumplan con los pagos por los servicios prestados, así se encuentren en proceso de liquidación
Atención de pacientes	a. Se afecta el cumplimiento de los atributos de la calidad en la atención de salud (acceso, oportunidad, pertinencia y continuidad) en cuanto al diagnóstico y el tratamiento de pacientes con cáncer. b. Disminución o concentración de la oferta del servicio de medicina nuclear y la atención integral del cáncer en el país c. Barreras en las dinámicas administrativas de las EPS para autorizaciones de tratamientos o de medicamentos necesarios como la tirotropina alfa

### Expertos de entidades reguladoras

Participaron 12 expertos de entidades que legislan, inspeccionan, vigilan y controlan el quehacer de los servicios de medicina nuclear en el país: el Ministerio de Salud y Protección Social, el Ministerio de Minas y Energía, el Servicio Geológico Colombiano, las secretarías departamentales de salud y la Asociación Colombiana de Medicina Nuclear.

En la primera ronda, el equipo investigador postuló cinco enunciados. Las respuestas y el grado de acuerdo de los participantes se presentan en el cuadro 4. En los enunciados 2, 3 y 5, se obtuvo un consenso de más del 80 %. En la segunda ronda de preguntas, se indagó sobre los enunciados en los que no hubo conceso durante la primera y se estructuró una sección de preguntas abiertas, con el fin de propiciar un mayor acuerdo en las conclusiones. Al final de la segunda ronda los expertos no lograron acuerdo frente a las afirmaciones 1 y 4, y expresaron opiniones divergentes sobre las regulaciones normativas exigidas a los servicios de medicina nuclear.

**Cuadro 4:** Resultados de los expertos reguladores en la primera y segunda rondas del método Delphi

Enunciado	Ronda 1			Ronda 2		
	Desacuerdo %	Ni acuerdo ni desacuerdo %	Acuerdo %	Desacuerdo %	Acuerdo &	
1. Las regulaciones actuales para los servicios de medicina nuclear están acordes con la realidad del Sistema General de Seguridad Social en Salud.	33,3	16,7	50,0	No	75,0	25,0
2. Las exigencias de los entes reguladores influyen en la prestación de los servicios de medicina nuclear para el control integral del cáncer.	8,0	^_^	92,0	SÍ	-	-
3. Los entes reguladores o los organismos de inspección, vigilancia y control deberían brindar asistencia técnica a los servicios de medicina nuclear, con el fin de dar cumplimiento a la normativa exigida y mantener la oferta de estos servicios en el territorio nacional.	16,7	^_^	83,4	SÍ	-	-
4. Existe coherencia y sinergia entre las normas emitidas por los entes reguladores y las exigencias de los organismos de inspección, vigilancia y control.	50,0	^_^	50,0	No	66,0	34,0
5. Los entes reguladores de los servicios de medicina nuclear deberían hacer veeduría sobre los organismos de inspección, vigilancia y control para verificar que la norma se interprete (aplique) correctamente.	^_^	^_^	100	SÍ	-	-

Escala de Likert: Acuerdo: totalmente de acuerdo (5) y de acuerdo (4); ni acuerdo ni descuerdo (3); desacuerdo: desacuerdo (2) y totalmente en desacuerdo (1)

La segunda sección del cuestionario incluía una serie de preguntas abiertas encaminadas a establecer las percepciones de los expertos frente a las consecuencias de las regulaciones normativas para los servicios de medicina nuclear. Durante la primera ronda, los expertos expresaron una pluralidad de opiniones, las cuales se resumieron por temas, y en la segunda ronda, se solicitó a los participantes que las enumeraran en orden de relevancia según su criterio. En el cuadro 5 se presentan las preguntas abiertas formuladas por los autores en la primera ronda, los temas surgidos de las declaraciones dadas por los expertos y el grado de relevancia valorado en la segunda ronda.

**Cuadro 5:** Calificación de la relevancia de las declaraciones de expertos en la segunda ronda

Pregunta	Temas de las declaraciones	Media	Moda	Relevancia
¿Qué medidas se podrían tomar para que, durante los procesos de habilitación, certificación y licenciamientos que apliquen a los servicios de medicina nuclear, los organismos de inspección, vigilancia y control se coordinen facilitando el cumplimento del prestador, con el fin de mantener la atención oportuna para los pacientes con cáncer en el país?	Delimitación de competencias de cada actuante	5,0	6	7
	Participación de médicos tratantes y miembros de la Asociación de Medicina Nuclear en la toma de decisiones normativas	4,2	1	5
	Divulgación y capacitación interdisciplinaria de organismos de inspección, vigilancia y control para unificar criterios	3,0	2	1
	Asistencias técnicas	4,0	6	4
	Exigencias de controles, calidad y listas de chequeo a los servicios	3,7	3	2
	Ajustar la regulación a la realidad socioeconómica del país	3,8	5	3
	Acciones coordinadas entre los organismos de inspección, vigilancia y control	4,3	4	6
¿Qué consecuencias podría traer para los servicios de medicina nuclear la modificación del Decreto 2106 de 2019 (Artículo 91)?	Demoras en la obtención de licencias por prolongación en los tiempos de espera en la habilitación de servicios oncológicos	2,4	1	2
	Cierre de servicios que hagan uso de equipos generadores de radiación ionizante	4,0	4	5
	Aumento de costos para los servicios debido a la necesidad de implementar nuevos procesos	2,2	2	1
	Afectación de la prestación de los servicios de medicina nuclear	3,0	3	3
	Demora en la obtención de licencias por falta de personal competente en las secretarías departamentales por desconocimiento de temas relacionados con la radiación	3,4	3	4
¿Qué consecuencias podría traer para los servicios de medicina nuclear la implementación de la Resolución 3100 de 2019?	La Resolución solicita estándares mínimos que no interfieren con el proceso de habilitación de servicios de medicina nuclear.	2,9	2	3
	La Resolución no diferencia los estándares para la administración de terapias de dosis alta y baja, por lo tanto, se pueden confundir los criterios y requisitos exigibles por parte de los auditores.	2,0	1	2
	La Resolución exige estándares de habilitación que requieren inversión en infraestructura.	1,9	1	1
	La Resolución traerá depuración de los servicios debido al fortalecimiento de grandes IPS y el cierre de pequeños prestadores.	3,2	4	4

## Discusión

La medicina nuclear está experimentando un crecimiento mundial debido al surgimiento de nuevos radiofármacos. Según cifras del OIEA, en el 2020, 134 de los 195 países contaban con servicios de medicina nuclear ^11^. Aunque en la región europea existen marcadas diferencias en el número total de procedimientos de medicina nuclear en los diferentes países (de 500 a 3.800 por millón de habitantes), el incremento generalizado de los procedimientos de medicina nuclear en la última década es un hecho. Por ejemplo, en Grecia, las salas para terapia de medicina nuclear con licencia de operación aumentaron en cerca del 60 % ^12^; en Latinoamérica y el Caribe, también ha habido un crecimiento significativo aunque heterogéneo en cuanto a la disponibilidad de la tecnología y los recursos humanos ^13^; por otra parte, durante la última década en Colombia la oferta de servicio de medicina nuclear, con y sin enfoque oncológico, ha disminuido levemente ^14^, lo que se debe principalmente a la apertura de servicios en el sector privado concentrada en los centros urbanos más grandes o en las ciudades capitales, situación que se repite en otros continentes ^7^.

Un factor que impacta la oferta es la gestión de los desechos radiactivos. Los servicios de medicina nuclear en los hospitales se han catalogado como una de las principales fuentes de radionúclidos en aguas residuales urbanas ^15^^,^^16^. Debido a la vida media relativamente larga de algunos radioisótopos, como el I-131, cuyo tiempo de semidesintegración física es de ocho días, los pacientes eliminan el radionúclido por medio de la orina y las excretas directamente a los sistemas de recolección de aguas y la red de alcantarillado. Con el aumento patente en la administración de la terapia con yodo radioactivo frente al diagnóstico de más casos subclínicos, esto se convierte en una preocupación para los hospitales con servicios de medicina nuclear ^17^.

Es imposible evitar la contaminación de los cuerpos de agua por fuentes radiactivas, por lo que se han promulgado normas para proteger la calidad de los recursos ambientales y la salud pública ^7^. A nivel mundial, las normas sobre los desechos radiactivos son heterogéneas y muchos países han aprobado regulaciones para la descarga directa de desechos líquidos hospitalarios en el sistema central de recolección de aguas residuales, lo que incluye desechos radiactivos, pero otros no ^17^.

Tal como lo señalan varios autores, países como Estados Unidos, Reino Unido, Dinamarca y Finlandia, no usan sistemas de decaimiento de la carga radiactiva y el I^131^ presente en las excretas de los pacientes hospitalizados se vierte de forma directa a la red de alcantarillado ^7^^,^^18^^-^^21^. Por el contrario, algunos países, entre ellos Alemania, Francia y España, han dispuesto que los tratamientos con gran actividad del I^131^ se realicen en áreas asistenciales equipadas con sistemas de recolección de aguas residuales ^22^^-^^24^, y, ciertamente, varios estudios han demostrado diferencias en los niveles de radionúclidos de origen médico en aguas residuales dependiendo del uso de sistemas de abatimiento radiactivo en los servicios de medicina nuclear ^17^.

En el presente estudio, se pudo determinar que una de las mayores dificultades que han tenido los servicios de medicina nuclear es la implementación del sistema de gestión de desechos radiactivos líquidos. Los participantes manifestaron que no hay claridad en la norma, lo que permite múltiples interpretaciones, y que no hay sinergia ni coherencia entre los organismos de inspección, vigilancia y control a nivel nacional.

Cabe resaltar que la mayoría de expertos clínicos consideran que la Resolución 180005 del 2010 y su modificación en el 2016 han contribuido a mejorar la calidad del agua de los ríos y grandes masas de agua, pese a que no todos los prestadores han adoptado un sistema de vertimientos. Sin embargo, en un estudio realizado por la dirección de asuntos nucleares del Servicio Geológico de Colombia en el 2020 para determinar la concentración y tasa de actividad del I^131^ en las principales entradas y salidas de una planta de tratamiento de aguas residuales en Bogotá, se evidenció que la concentración radioquímica era mayor al valor de referencia para agua potable y estaba cerca del límite de descarga durante el día. Esta es la primera investigación de este tipo en el país, y deben hacerse más estudios para evaluar estos resultados en otras regiones del país ^25^.

Las divergencias de normativa entre países obedecen a su inclusión en el OIEA, pues todos los países que se acogen a sus recomendaciones han tenido que ajustar la legislación sobre los servicios de medicina nuclear y estos, a su vez, se han visto obligados a modificar sus protocolos, instalaciones y modelos de prestación para cumplir con la norma. La adaptación de las recomendaciones internacionales a la legislación nacional no ha sido fácil; la experiencia local es equiparable a la de países europeos como Italia, donde ha habido cierta complejidad y falta de coherencia en la legislación frente al manejo de residuos médicos radiactivos, por lo que se ha exigido que los requisitos sean más sencillos, coherentes y eficaces ^26^.

Lograr que la normativa sea coherente con el funcionamiento de los servicios y se ajuste a las condiciones locales, es un reto que debe abordarse para mejorar el desarrollo de la medicina nuclear en el país. En la Resolución 3100 del 2019 del Ministerio de Salud y Protección Social, se definen los procedimientos y condiciones para la habilitación de los servicios de salud, y en la Resolución 4245 del 2015, se establecen los requisitos para obtener la certificación en buenas prácticas de elaboración de radiofármacos y la lista las condiciones que debe cumplir la radiofarmacia según el nivel de complejidad ^27^^,^^28^. El cumplimiento de la normativa implica, en ocasiones, la modificación de la planta física de la institución o servicio. Varios de los expertos participantes en este estudio manifestaron que los servicios de medicina nuclear no podían acondicionar su planta física para cumplir con los requisitos de habilitación nacional debido al costo. Esta compleja situación se replica en muchos países, por ejemplo, en Indonesia solo 10 de sus 17 servicios de medicina nuclear están funcionando debido a que no han podido cumplir los requisitos de infraestructura establecidos por el Ministerio de Salud para la administración de radioisótopos ^29^. El diseño ajustado a los objetivos de los centros médicos y hospitalarios necesita una adecuada planeación desde el principio de la construcción y la participación de físicos médicos, radioquímicos médicos y arquitectos ^30^.

El desarrollo reciente de dispositivos y técnicas para el tratamiento de la orina y las excretas de pacientes tratados con I^131^ más fáciles de instalar y menos costosos, aparece como una solución frente a algunos de los problemas descritos por los participantes del estudio ^31^^-^^33^. Es fundamental seguir buscando alternativas de tratamiento para los desechos radiactivos médicos con I^131^ que no afecten la oferta del servicio y que, por el contrario, permitan una descontaminación adecuada bajo condiciones de seguridad tanto para el personal médico como para los pacientes.

Dada la inversión que deben realizar los hospitales y centros de salud para la atención de pacientes oncológicos y la elevada demanda de tratamientos oncológicos con yodo radioactivo, en el Foro Económico Mundial del 2010 se evidenció que los servicios oncológicos tienen gastos por encima de sus ganancias ^34^, tal como quedó demostrado en un estudio en Brasil en que se concluyó que la oferta de terapias con I^131^ a pacientes que requieren hospitalización implicaba mayores gastos ^35^. En la mayoría de países que se acogen a las recomendaciones del OIEA, la administración de terapias de I^131^ con actividad superior a 30 mCi (1.110 mbBq) para el manejo del cáncer de tiroides, se hospitaliza a los pacientes en salas de aislamiento con manejo de desechos radiactivos hasta alcanzar la dosis requerida para ser dados de alta, pero esto puede tardar un poco más de 24 horas, lo que incrementa los costos del servicio. Por ello, en países como Brasil, Chile o Colombia, son pocos los hospitales públicos que disponen de tratamientos para el cáncer de tiroides con altas dosis de I-131 ^36^. También en Japón, la estricta regulación ha llevado a una limitación en la oferta de la terapia con yodo radiactivo, lo que ha prolongado los tiempos de espera para recibir tratamientos con I-131 y otros radiofármacos ^37^. Para evitar el incumplimiento de las normas, en algunos países los entes gubernamentales son los encargados de brindar los servicios de medicina nuclear. Por ejemplo, en Pakistán, la mayor oferta de servicios de medicina nuclear la brinda la Comisión de Energía Atómica y el tratamiento ambulatorio con yodo radiactivo está más disponible en todo el país. Las instalaciones para pacientes hospitalizados que requieren dosis altas son limitadas, pero dada la distribución de los centros en todo el país, se cubre una gran parte del territorio ^38^.

En definitiva, los problemas derivados de la normativa son parecidos en muchos países, pero también las perspectivas de progreso en este campo. La oncología nuclear crecerá aún más a medida que se desarrollen nuevas terapias con radioisótopos médicos ^39^, los cuales potenciarán la denominada medicina personalizada centrada en el paciente ^40^. Así, el futuro de la medicina nuclear se centra en la producción y la aplicación de radioisótopos para la terapia dirigida y la medicina personalizada ^41^^,^^42^, con lo que, además, se incrementará la necesidad de especialistas bien capacitados, capaces de gestionar aspectos tecnológicos de la disciplina y las terapias oncológicas innovadoras en un entorno multidisciplinario ^40^.

En tanto los logros de la medicina nuclear aumenten, la gestión de los desechos radiactivos también mejorará. En este sentido, se necesitará el apoyo de organizaciones internacionales como el OIEA mediante proyectos nacionales y regionales, así como alianzas público-privadas y el compromiso gubernamental para desarrollar capacidades en torno a la práctica de la medicina nuclear.

En conclusión, los hallazgos del estudio sugieren que los servicios de medicina nuclear en Colombia atraviesan un momento de muchos desafíos institucionales, normativos y económicos, que ponen en riesgo el desarrollo y mantenimiento de los servicios de medicina nuclear para la atención oncológica, y, en consecuencia, el control integral del cáncer en el país. Este panorama reclama esfuerzos inmediatos de articulación de todos los sectores involucrados a nivel nacional e internacional. Se requiere del acompañamiento constante de los organismos encargados de velar por el cumplimento de la norma, procurando que sea coherente con las condiciones del país, el sistema de salud y los servicios, y siempre en beneficio de los pacientes.
